# The comorbidity of borderline personality disorder and posttraumatic stress disorder: revisiting the prevalence and associations in a general population sample

**DOI:** 10.1186/s40479-015-0032-y

**Published:** 2015-07-24

**Authors:** Emily M. Scheiderer, Phillip K. Wood, Timothy J. Trull

**Affiliations:** 104 Psychology Building, University of Missouri – Columbia, Columbia, MO 65211 USA; 210 McAlester Hall, University of Missouri – Columbia, Columbia, MO 65211 USA

**Keywords:** Borderline personality disorder, Posttraumatic stress disorder, Comorbidity, Childhood sexual abuse, General population

## Abstract

**Background:**

The comorbidity of borderline personality disorder (BPD) and posttraumatic stress disorder (PTSD) is frequent, yet not well understood. The influence of childhood sexual abuse (CSA) in the development of this comorbidity has been a focus of prior clinical studies, but empirical evidence to generalize this focus to the broader population is lacking. Primary aims of the present study included evaluation of: (a) the association of this comorbidity with decrements in health-related quality of life (HRQOL) and (b) the importance of CSA as a predictive factor for this comorbidity in a general population sample.

**Methods:**

We utilized data from Wave 2 of the *National Epidemiological Survey on Alcohol and Related Conditions*, a nationally representative face-to-face survey evaluating mental health in the non-institutionalized adult population of the United States. Data from respondents who met criteria for BPD and/or PTSD were analyzed (*N* = 4104) to assess potential associations between and among lifetime BPD-PTSD comorbidity, CSA, gender, healthcare usage, and mental and physical HRQOL.

**Results:**

Lifetime comorbidity of BPD and PTSD was associated with more dysfunction than either individual disorder; and the factors of gender, age, and CSA exhibited significant effects in the prediction of this comorbidity and associated decrements in HRQOL.

**Conclusions:**

Results support the measured focus on CSA as an important, but not necessary, etiologic factor and emphasize this comorbidity as a source of greater suffering and public health burden than either BPD or PTSD alone. The differential impact of these disorders occurring alone versus in comorbid form highlights the importance of diagnosing both BPD and PTSD and attending to lifetime comorbidity.

Borderline personality disorder (BPD) and posttraumatic stress disorder (PTSD) co-occur in ways that are not yet well understood, especially outside of clinical samples. Fundamental questions remain, for example, regarding whether these disorders share common underlying factors, whether one disorder predisposes the other and, more fundamentally, whether BPD and PTSD are two independent disorders or variants of a single syndrome on a spectrum of trauma-related pathology (see, e.g., [1]).

BPD-PTSD comorbidity rates vary across studies, likely reflecting differences in implementation of diagnostic criteria, methods of sampling and assessment, and management of extraneous variables (e.g., demographics and other comorbidities). Studies employing treatment-seeking clinical samples report rates of PTSD among individuals with BPD ranging from 25 to 58 %, and rates of BPD among individuals with PTSD ranging from 10 to 76 % (see [2]). Recent community-based, epidemiological studies report prevalence rates of comorbid PTSD among those meeting diagnostic criteria for BPD ranging from 30 to 50 % (e.g., [2–4]), though some report lower numbers (e.g., 17 % [5]); and rates of comorbid BPD among those with PTSD ranging from 6.6 to 24 % [2, 5–7]. Additionally, odds ratios for the association between BPD and PTSD reflect high comorbidity, with BPD groups having 7–10 times the odds of co-occurring PTSD [3, 8].

## Comparing BPD and PTSD

As defined by the *Diagnostic and Statistical Manual of Mental Disorders* (*DSM-IV-TR* [9] and its recent revision, *DSM-5* [10]), BPD and PTSD appear quite different, save for the similarity that both may include dissociative symptoms. Recently, Frías and Palma [1] summarized the overlap in psychopathological mechanisms of BPD and PTSD by referencing empirical support for the characterization that both disorders exhibit more dissociative symptoms than (a) healthy controls and/or (b) other mental disorders. Despite what seems to be a narrow phenomenological overlap between BPD and PTSD, the clinical presentation of these two disorders can be easily confused [11, 12]. Furthermore, influential authors on the topic, Herman and van der Kolk [13], described apparent similarities of disturbance across BPD and PTSD in the core domains of affect regulation, impulse control, reality testing, interpersonal relationships, and self-integration.

Related to the question of similarity among BPD and PTSD symptoms, the question of potential interaction of symptoms across these disorders also eludes a simple answer. Evidence from a variety of clinical studies supports the conceptualization of BPD and PTSD as distinct clinical syndromes that exhibit relatively independent constellations of symptoms (e.g., [14–16]). Findings from other clinical studies, however, suggest that the impact of this comorbidity may include exacerbation of core symptoms of these two disorders. Particularly, whereas some findings suggest lesser or similar levels of emotion dysregulation-related symptoms among individuals with this comorbidity versus BPD only [17, 18], other findings suggest that comorbid PTSD may play an exacerbating role in the expression of affective instability [19, 20], and on the lethality, intent, and triggers for intentional self-injury [21] in BPD.

### Etiology and the potential role of childhood sexual abuse

Common etiologic factors may play a role in the co-occurrence of these two disorders. For decades, discussion of etiological overlap between BPD and PTSD has focused on childhood trauma, and particularly, childhood sexual abuse (CSA). Clearly, CSA can fulfill the required traumatic event criterion for diagnosis of PTSD (e.g., ‘Criterion A’ of the *DSM-IV* and *DSM-5* [9, 10]). With regard to BPD, multidimensional theories recognize CSA as a significant psychological risk factor (e.g., [11, 22, 23]). Numerous studies report higher rates of CSA in BPD patient samples than in other, ‘near-neighbor’ diagnosis samples (e.g., [24–29]). Moreover, examination of the differential predictive relationships of childhood physical and sexual abuse with later BPD diagnosis suggests that CSA may be uniquely associated with BPD (e.g., [22, 30, 31]). The extent to which CSA interacts with other risk factors for BPD may contribute to this CSA-BPD association. For example, CSA may interact with pathological family environment—with disrupted family dynamics in cases of incest, or with family neglect in cases of extra-familial CSA—and may be one of the clearest examples of the extreme invalidation theorized to be a key early-life factor in the development of BPD [22].

Referencing the high prevalence of childhood trauma, particularly CSA, in BPD samples, as well as anecdotal clinical experience, some suggest that BPD is a chronic variant of PTSD (e.g., [13, 32]). However, findings, such as those of Zanarini et al. [33], in which substantially less than 100 % of a high-severity, inpatient BPD sample reported lifetime history of PTSD, argue against this conceptualization. Rather, such findings support the diathesis-stress and multifactorial models of BPD etiology, in which different combinations and proportions of inborn temperamental vulnerability and life experiences result in BPD. As many researchers investigating childhood abuse in BPD samples note, the role of CSA in the pathogenesis of BPD, though important, is neither specific nor sufficient for the development of BPD (e.g., [23, 34–38]).

### Clinical and public health outcomes

The comorbidity of BPD and PTSD may be an important factor in clinical course, treatment response, and health-related quality of life in BPD and PTSD samples. The association of this comorbidity with particular difficulties for affected individuals, treatment providers, and society (e.g., public health burden) and the body of evidence suggesting that this comorbidity may affect expression of core features of these disorders, together, underscore the need to consider this comorbidity in research, treatment, and policy decisions. Better understanding of this comorbidity could lead to more specific and promising treatment approaches [39]. Recent development of the first fully integrated, concurrent, and empirically supported treatment for comorbid BPD and PTSD by Harned and colleagues [40, 41] reflects progress in this direction.

Few studies have examined BPD-PTSD comorbidity in the general population. One such study by Connor et al. [42] contributed preliminary support for the association of greater burden of distress and dysfunction with this comorbidity in the general population, but suffered limited generalizability due to its regionally bound sample, very small comorbid group, lack of BPD-only group, and use of posttraumatic stress symptoms (PTSS) rather than full PTSD. Pagura et al. [2] presented the first examination of BPD-PTSD comorbidity in a large, nationally representative sample: Data from Wave 2 of the *National Epidemiological Survey on Alcohol and Related Conditions* (NESARC [43]) were used to examine differences in psychopathology, childhood traumatic events, and health-related quality of life (HRQOL) across individuals with BPD, PTSD, and comorbid BPD-PTSD. Results suggested that individuals with the comorbid diagnosis had poorer HRQOL, more Axis I comorbidity, increased odds of lifetime suicide attempt, and higher prevalence of repeated traumatic events in childhood than individuals with either diagnosis alone [2]. Individuals with the comorbidity had significantly lower mental HRQOL scores than those with BPD-only or PTSD-only; and individuals with PTSD-only and those with the comorbidity had significantly lower physical HRQOL scores than those with BPD-only [2]. Additionally, examining what Pagura et al. refer to as symptom severity, those in the comorbid group endorsed significantly more BPD and PTSD symptom items than those in the BPD-only and PTSD-only groups.

In their assessment of PD diagnoses, however, Pagura et al. [2] employed diagnostic rules that do not reflect the current consensus among PD researchers and clinicians [44]. Most widely accepted PD diagnosis guidelines (e.g., [10]) stipulate that each criterion causes significant distress and/or impairment in order to count toward the diagnosis. The guidelines used by Pagura et al. only required distress and/or impairment associated with one of the requisite criteria in order to achieve the diagnosis, resulting in potential over-diagnosis and prevalence rates that were considerably higher than those seen in other community studies [44].

### Present study

The present study aimed to further examine BPD-PTSDcomorbidity in a large, representative community sample, utilizing data from Wave 2 of the *National Epidemiological Survey on Alcohol and Related Conditions* (NESARC [43]) to assess potential associations between and among BPD-PTSD comorbidity, CSA, gender, healthcare usage, and mental and physical health-related quality of life (HRQOL). Diverging from the prior NESARC-based study by Pagura et al. [2], the present study employed Trull et al.’s [44] re-analysis of the NESARC PD data, which, in line with the predominant view on PD diagnosis, required that each criterion be associated with distress/impairment in order to count toward the diagnosis. Additionally, the present study examined predictive factors and patterns of healthcare usage across the BPD-only, PTSD-only, and comorbid groups; focused on the prevalence of a broad array of traumatic events across the diagnostic groups; investigated the contribution of CSA, itself, to variance in HRQOL; used lifetime, rather than past-year, diagnosis of PTSD to examine effects of lifetime comorbidity on public health-related outcomes; and statistically examined the distinction between the additive and synergistic effects of BPD and PTSD on these public health-related outcomes.

We hypothesized that: (1) CSA would be more prevalent among both men and women with the comorbidity than among those with one of the two diagnoses; and among men and women with BPD-PTSD comorbidity, CSA would be among the most prevalent types of traumatic experience. (2) Positive history of CSA would be associated with greater odds of meeting diagnostic criteria for BPD-PTSD comorbidity than meeting diagnostic criteria for either PTSD or BPD alone. (3) There may be a gender difference in the association of BPD with PTSD. Specifically, the odds ratios for prediction of BPD-PTSD comorbidity may be different for women and men; (4) BPD-PTSD comorbidity would be associated with worse mental and physical HRQOL and with greater and more intensive use of health care services than BPD or PTSD alone. And (5) CSA would be associated with worse mental and physical HRQOL, but this association would not be as robust as that with BPD-PTSD comorbidity.

## Method

The *National Epidemiologic Survey on Alcohol and Related Conditions* (NESARC) was a nationally representative face-to-face survey evaluating mental health in the non-institutionalized adult (age 18 and over) population of the United States. NESARC psychiatric diagnoses were assessed by trained lay interviewers using a fully structured interview. Wave 2, which included both BPD and PTSD assessments, was conducted in 2004-2005 with a sample of 34,653 completed interviews [43]. Oversampling of African-Americans, Latinos, and young adults (ages 18-24) was implemented. The data were weighted according to this oversampling and to reflect design characteristics of the survey. Adjustments were made for nonresponse across sociodemographic characteristics; and the weighted Wave 2 data were then adjusted, based on the 2000 Decennial Census, to represent the civilian population on sociodemographic variables including region, age, race, and gender [3].

### Measures

#### *DSM-IV* diagnoses

BPD was assessed on a lifetime basis using the Alcohol Use Disorder and Associated Disabilities Interview Schedule—*DSM-IV* Version (AUDADIS-IV [45]). PTSD was assessed with regard to past-year and prior-to-past-year diagnoses, also using the AUDADIS-IV. The AUDADIS-IV is a fully structured diagnostic interview designed to assess alcohol, drug, and other mental disorders in both general and clinical populations according to DSM-IV criteria [45]. The AUDADIS-IV requires that personality disorder symptoms should occur “most of the time throughout your life, regardless of the situation and who you were with.” Using a subsample of 1899 respondents, fair to good test-retest and inter-rater reliability have been demonstrated for Wave 2 Axis I and II AUDADIS-IV diagnoses [46, 47]. Kappas indicated fair to good agreement: For PTSD past-year and lifetime diagnoses, kappas were 0.77 and 0.64, respectively; and for BPD, 0.71. Internal consistency of symptom scales associated with BPD and PTSD fell within the good range (alpha = 0.75–0.89); and reliability of risk factor measures fell in the good-to-excellent range (intraclass correlations = 0.50–0.94; alpha = 0.64–0.90), further indicating the usefulness of the AUDADIS-IV diagnostic measures [47].

The AUDADIS-IV PTSD section began with an enumeration of 27 types of potentially traumatic events designed to operationalize part 1 of *DSM-IV* Criterion A. Respondents who endorsed more than one potentially traumatic event were asked to indicate which one they considered to be the “worst stressful event.” Respondents were then asked whether they felt extremely frightened, helpless, or horrified during the event (consistent with part 2 of *DSM-IV* Criterion A), as well as whether they felt that they or someone close to them might die, be seriously injured, or disabled during the event. Subsequent items assessed *DSM-IV* PTSD symptoms—criteria B through D—and duration (of at least one month; criterion E) in reference to the respondents’ indicated event. At least one symptom within Criterion B (re-experiencing), at least three within Criterion C (avoidance and numbing), and at least two within Criterion D (hyperarousal), together with fulfillment of the *DSM-IV* clinical significance criterion of impairment or distress, were required for diagnosis of lifetime PTSD (see, e.g., [7]).

In the present study, lifetime diagnosis of PTSD was used in all central analyses. However, to offer findings that would be more comparable with past (e.g., [2, 14, 16]) and future studies using current, or past-year, PTSD in analyses of HRQOL and healthcare usage, we also report supplementary analyses based on current PTSD diagnosis below. With regard to diagnosis of BPD, the present study employed Trull et al.’s [44] re-analysis of the NESARC PD data, requiring that each criterion be associated with distress/impairment in order to count toward the diagnosis. This re-analysis significantly reduces the PD prevalence rates, bringing them much more into line with recent epidemiological studies in the U.S. and Britain.

#### Childhood sexual abuse

History of childhood sexual abuse (CSA) was assessed based on response to the NESARC interview item, “Were you ever sexually assaulted, molested, or raped, or did you ever experience unwanted sexual activity?” along with the following item assessing age at onset. Endorsement of the former, together with an answer less than 16 (years of age) on the latter, counted as positive endorsement of CSA (see [2]).

#### Other traumatic experiences

Traumatic experiences, other than CSA, assessed in the NESARC interview and utilized in the present study include those related to: military combat, peacekeeping, civilian experience of war, experience as a refugee, life-threatening accident/illness, natural disaster, physical assault/abuse, neglect, witnessing serious fights at home, kidnapping/being held hostage, being stalked, being held up with a weapon, experiences of terrorist attack, witnessing severe injury/death, unexpected death of someone close, and serious illness/injury/traumatic experience of someone close.

#### Physical and mental HRQOL

The SF-12v2 Health Survey [48] was used to assess participants’ physical and mental HRQOL. Eight scales, four physical (General Health, Physical Functioning, Role Physical, and Bodily Pain) and four mental (Mental Health, Social Functioning, Role Emotional, and Vitality), comprise this 12-item short-form health survey. The SF-12v2 (standard version) asks respondents to recall over the last four weeks. For example, “During the past 4 weeks, how much of the time has your physical health or emotional problems interfered with your social activities like visiting with friends, relatives, and so forth?” Scoring was conducted based on the SF-12v2 user’s manual [49], resulting in norm-based scores with a standardized range (0 to 100) and mean (50).

#### Healthcare usage

NESARC interview items assessing number of overnight hospitalizations, days spent in the hospital, and number of times treated in a hospital emergency room in the last 12 months were used to examine aspects of general healthcare usage.

### Analyses

Data analyses were conducted in SAS/STAT® software, version 9.2 (Copyright, SAS Institute, Inc., registered trademark of SAS Institute, Inc., Cary, NC, USA), and Mplus, and the Wave 2 stratification and weighting systems that were part of the NESARC’s complex survey design were employed in these analyses. In SAS 9.2, such complex survey sample analyses required use of procedures equipped specifically for incorporation of complex sampling design (i.e., PROC SURVEYMEANS, PROC SURVEYREG, PROC SURVEYFREQ). Due to the number of analyses and large sample employed, the *p-*value of less than .01, rather than .05, was used to assess statistical significance of results.

## Results

### Demographic characteristics

Results from descriptive analyses of diagnostic status and sociodemographic characteristics are presented in Table 1. A total of 3074 individuals met criteria for the PTSD-only group, 483 met for the BPD-only group, and 547 for the comorbid group, comprising the full subsample of NESARC Wave 2 respondents (*N* = 4104) whose data were examined in the present study. These numbers indicate the following comorbidity rates: 53.11 % of those who met criteria for BPD also met criteria for lifetime PTSD; and 14.69 % of those who met for PTSD also met for BPD. Results of the Wald chi-square tests for independence of the row and column variables in the two-way tables crossing the diagnostic group variable (BPD-only vs. PTSD-only vs. Comorbid BPD-PTSD) with each of the sociodemographic variables (age group, gender, U.S. region, marital status, education level, household income, and race/ethnicity) indicated that the groups differed in terms of age (*χ*^2^[6, *n* = 4104] = 122.21, Wald F*[6, 3687] = 20.34, *p* < .0001), gender (*χ*^2^[2, *n* = 4104] = 47.47, Wald F*[2, 3691] = 23.73, *p* < .0001), marital status (*χ*^2^[4, *n* = 4104] = 45.83, Wald F*[4, 3689] = 11.45, *p* < .0001), and household income (*χ*^2^[6, *n* = 4104] = 35.84, Wald F*[6, 3687] = 5.97, *p* < .0001).

**Table 1:** Demographic characteristics of the sample

Characteristics	PTSD-only	BPD-only	Comorbid
	(*n* = 3074)	(*n* = 483)	(*n* = 547)
	*n* (column %, row %)	*n* (column %, row %)	*n* (column %, row %)
*Gender*			
Male	815 (26.5, 67.8)	224 (46.4, 18.7)	162 (29.6, 13.5)
Female	2259 (73.5, 77.8)	259 (53.6, 8.9)	385 (70.4, 13.3)
*Age*			
20–29	381 (12.4, 63.2)	111 (23.0, 18.4)	111 (20.3, 18.4)
30–44	1007 (32.8, 72.0)	181 (37.5, 12.9)	211 (38.6, 15.1)
45–64	1246 (40.5, 77.6)	156 (32.3, 9.7)	203 (37.1, 12.6)
65+	440 (14.3, 88.5)	35 (7.2, 7.0)	22 (4.0, 4.4)
*Race/Ethnicity*			
African American	661 (21.5, 75.1)	98 (8.8, 11.4)	121 (22.1, 13.8)
American Indian/Alaskan	68 (2.2, 68.0)	11 (2.3, 11.0)	21 (3.8, 21.0)
Asian/Pacific Islander	66 (2.1, 84.6)	9 (1.9, 11.5)	3 (0.5, 3.9)
Caucasian	1774 (57.7, 75.4)	288 (59.6,12.2)	290 (53.0, 12.3)
Hispanic	505 (16.4, 72.8)	77 (15.9, 11.1)	112 (20.5, 16.1)
*Education*			
< High school	538 (17.5, 74.0)	82 (17.0, 11.3)	107 (19.6, 14.7)
High school or equivalent	806 (26.2, 72.0)	156 (32.3, 13.9)	158 (28.9, 14.1)
Some college+	1730 (56.3, 76.7)	245 (50.7, 10.9)	282 (51.6, 12.5)
*Marital status*			
Married/cohabiting	1483 (48.2, 78.6)	197 (40.8, 10.4)	206 (37.7, 10.9)
Widowed/separated/divorced	1049 (34.1, 74.6)	159 (32.9, 11.3)	199 (36.4, 14.1)
Never married	542 (17.6, 66.8)	127 (26.3, 15.7)	142 (26.0, 17.5)
*Household income*			
$0–$19,999	890 (29.0, 70.7)	133 (27.5, 10.6)	236 (43.1, 18.7)
$20,000–$34,999	677 (22.0, 75.3)	106 (21.9, 11.8)	116 (21.2, 12.9)
$35,000–$59,999	681 (22.2, 73.8)	133 (27.5, 14.4)	109 (19.9, 11.8)
$60,000+	826 (26.9, 80.7)	111 (23.0, 10.9)	86 (15.7, 8.4)
*U.S. region*			
New England	134 (4.4, 74.9)	15 (3.1, 8.4)	30 (5.5, 16.8)
Mid Atlantic	414 (13.5, 74.6)	63 (13.0, 11.4)	78 (14.3, 14.1)
East North Central	419 (13.6, 73.6)	60 (12.4, 10.5)	90 (16.5, 15.8)
West North Central	173 (5.6, 80.1)	21 (4.3, 9.7)	22 (4.0, 10.2)
South Atlantic	643 (20.9, 75.8)	103 (21.3, 12.1)	102 (18.6, 12.0)
East South Central	212 (6.9, 75.7)	25 (5.2, 8.9)	43 (7.9, 15.4)
West South Central	294 (9.6, 72.8)	52 (10.8, 12.9)	58 (10.6, 14.4)
Mountain	230 (7.5, 75.2)	45 (9.3, 14.7)	31 (5.7, 10.1)
Pacific	555 (18.1, 74.3)	99 (20.5, 13.3)	93 (17.0, 12.4)

Looking at the descriptive statistics presented in Table 1, several patterns stand out: Women tended to be overrepresented in the PTSD-only group and men in the BPD-only group. The BPD-only and comorbid groups tended to be overrepresented in the ‘never married’ category, whereas PTSD-only was overrepresented in the ‘married or living with someone as if married’ category. The PTSD-only group was overrepresented at the higher levels of education (though the Wald’s Chi-square test indicated that the overall group differences for education level were not significantly different from chance) and household income; whereas the comorbid group was overrepresented at the lowest levels of these. In terms of race/ethnicity, PTSD-only was slightly underrepresented and the comorbidity was overrepresented in the American Indian group. Nearly the opposite was true for the Asian group, in which PTSD-only was overrepresented and the comorbidity was underrepresented. In the Hispanic group, the comorbidity was slightly overrepresented as compared to the two single-disorder diagnoses.

### CSA and other trauma prevalence rates

Calculation of the prevalence rates of different traumatic experiences across the diagnostic groups and genders (Table 2) supported the hypothesis that CSA would be more prevalent among women and men with the comorbidity than those with either individual disorder, and that CSA would be one of the most prevalent trauma types among women and men with the comorbidity. Approximately 36 % (196 of 547) of those in the comorbid group, 18.43 % (89 of 483) of those in the BPD-only group, and 19.52 % (600 of 3074) of those in the PTSD-only group reported CSA. Whereas the CSA prevalence rate for women was slightly more than double that for men in the comorbid group (43.42 % vs. 19.14 %), this gender difference was closer to 4-fold in the BPD-only (28.24 % vs. 7.62 %) and PTSD-only (24.33 % vs. 6.51 %) groups. For both women and men, the comorbid group had close to double the CSA prevalence rate seen in either the BPD-only or the PTSD-only group. A Wald’s Chi-square test indicated that the difference in CSA prevalence across the diagnostic groups was significant (*χ*^2^[2, *n* = 4082] = 35.7594, Wald F*[2, 3669] = 17.87, *p* < .0001). Looking at the full range of traumatic experience prevalence rates, CSA was the 6^th^ most prevalent traumatic experience, out of 23 different types of traumatic experiences, in the comorbid group. In the BPD-only group, CSA ranked 10^th^; and in the PTSD-only group CSA ranked 9^th^ most prevalent. These analyses addressed all traumatic events reported, and as such, were not limited to those events indicated as the ‘worst stressful event.’

**Table 2:** Prevalence of different types of traumatic experience across the diagnostic groups

	PTSD-only	BPD-only	Comorbid
	(*n* = 3074)	(*n* = 483)	(*n* = 547)
	*n* (% within dx group, prevalence rank within dx group)	*n* (% within dx group, prevalence rank within dx group)	*n* (% within dx group, prevalence rank within dx group)
Childhood sexual abuse/assault (CSA)	600 (19.52 %, 9)	89 (18.43 %, 10)	196 (35.83 %, 6)
Adult sexual abuse/assault (ASA)	175 (5.69 %, 16)	17 (3.52 %, 15)	39 (7.13 %, 16)
Military combat	178 (5.79 %, 15)	12 (2.48 %, 17)	26 (4.75 %, 17)
Peacekeeping/relief work in war zone	58 (1.89 %, 21)	13 (2.69 %, 16)	11 (2.01 %, 19)
Civilian experience of war	92 (2.99 %, 18)	8 (1.66 %, 18)	15 (2.74 %, 18)
Refugee experience	59 (1.92 %, 20)	1 (0.21 %, 20)	8 (1.46 %, 20)
Serious/life-threatening accident	782 (25.44 %, 5)	151 (31.26 %, 3)	201 (36.75 %, 5)
Serious/life-threatening illness	834 (27.13 %, 3)	123 (25.47 %, 6)	187 (34.19 %, 8)
Natural disaster	686 (22.32 %, 7)	115 (23.81 %, 7)	150 (27.24 %, 10)
Physical attack/abuse by caregiver before age 18	351 (11.42 %, 13)	57 (11.80 %, 12)	139 (25.41 %, 12)
Neglect by caregiver before age 18	313 (10.18 %, 14)	55 (11.39 %, 13)	138 (25.23 %, 13)
Witnessed fights between caregivers before age 18	787 (25.60 %, 4)	141 (29.19 %, 4)	224 (40.95 %, 3)
Intimate partner violence	683 (22.22 %, 8)	104 (21.53 %, 9)	223 (40.77 %, 4)
Physical attack by anyone else	467 (15.19 %, 11)	113 (23.40 %, 8)	166 (30.35 %, 9)
Kidnapped/held hostage/POW	104 (3.38 %, 17)	13 (2.69 %, 16)	45 (8.23 %, 15)
Stalked	585 (19.03 %, 10)	81 (16.77 %, 11)	142 (25.96 %, 11)
Mugged	700 (22.77 %, 6)	138 (28.57 %, 5)	192 (35.10 %, 7)
Death of someone close in a terrorist attack	58 (1.89 %, 21)	5 (1.04 %, 19)	8 (1.46 %, 20)
Injured in terrorist attack	7 (0.23 %, 22)	0 (0.0 %, 21)	1 (0.18 %, 22)
Direct experience of terrorist attack (without injury)	63 (2.05 %, 19)	8 (1.66 %, 18)	5 (0.19 %, 21)
Saw someone badly injured/killed	1148 (37.35 %, 2)	164 (33.95 %, 2)	257 (46.98 %, 2)
Unexpected death of someone close	1937 (63.01 %, 1)	269 (55.69 %, 1)	386 (70.57 %, 1)
Any other traumatic experience	358 (11.65 %, 12)	39 (8.07 %, 14)	100 (18.28 %, 14)

### Predicting BPD-PTSD comorbidity: odds ratios based on CSA, age, and gender

Results from the logistic regressions of diagnostic status on CSA status, age group, and gender indicated that these predictors had differential associations with diagnostic status, that some gender differences occurred in the prediction, and that some predictors were operating differently across the genders. Specifically, the hypothesis that positive history of CSA would be associated with greater odds of meeting diagnostic criteria for BPD-PTSD comorbidity than meeting diagnostic criteria for either PTSD or BPD alone was supported: Those who reported history of CSA (vs. those who did not) had significantly greater odds of being in the comorbid group than in either the BPD-only (*OR*_*comorbid v. BPD*_ = 2.259, 99 % CI [1.373, 3.717]) or the PTSD-only group (*OR*_*comorbid v. PTSD*_ = 2.359, 99 % CI [1.681, 3.310]).

Results partially supported the hypothesis of gender difference in the association of BPD with PTSD: Being a woman significantly increased the odds of being in the PTSD-only group as compared to the BPD-only group (*OR*_*PTSD v. BPD*_ = 2.549, 99 % CI [1.791, 3.628]) or the comorbid group (*OR*_*PTSD v. comorbid*_ = 1.580, 99 % CI [1.124, 2.222]), and being a woman increased the odds of being in the comorbid group as compared to the BPD-only group (*OR*_*comorbid v. BPD*_ = 1.613, 99 % CI [1.044, 2.493]). Conversely, being a man significantly increased the odds of being in the BPD-only group as compared to the comorbid group (*OR*_*BPD v. comorbid*_ = 1.613, 99 % CI [1.044, 2.493]), significantly increased the odds of being in the BPD-only group as compared to the PTSD-only group (*OR*_*BPD v. PTSD*_ = 2.549, 99 % CI [1.791, 3.628]), and significantly increased the odds of being in the comorbid group as compared to the PTSD-only group (*OR*_*comorbid v. PTSD*_ = 1.580, 99 % CI [1.124, 2.222]). Conducting these logistic regressions separately for women and men yielded a pattern of odds ratios that was very similar across the genders, with two notable exceptions: (1) Among women, older age conferred slightly greater odds of being in the comorbid group than in the BPD-only group (*OR*_*comorbid v. BPD*_ = 1.236, 95 % CI [1.013, 1.509]); whereas in men, this older age-comorbidity association was not indicated (*OR*_*comorbid v. BPD*_ = 1.005, 95 % CI [0.756, 1.335]). (2) For men with CSA, the odds ratio for having the comorbid diagnosis versus PTSD-only was even greater (approximately 3-fold: *OR*_*comorbid v. PTSD*_ = 3.176, 99 % CI [1.366, 7.383]) than that for women with CSA (approximately 2-fold: *OR*_*comorbid v. PTSD*_ = 2.207, 99 % CI [1.507, 3.231]). Additionally, with regard to age as a predictor across the whole sample (i.e., not separating men and women), those in the older age groups (as compared to the younger age groups) had significantly greater odds of being in the PTSD-only group than in the comorbid group (*OR*_*PTSD v. comorbid*_ = 1.493, 99 % CI [1.255, 1.776]) or in the BPD-only group (*OR*_*PTSD v. BPD*_ = 1.664, 99 % CI [1.399, 1.979]).

### Health-Related Quality of Life (HRQOL)

#### Main effects of diagnostic group

Results from the series of SAS SURVEYREG procedures conducted to assess the variance in scores on each SF-12v2 scale accounted for by diagnostic status (BPD-only, PTSD-only, or comorbid), by gender, and by their interaction (reported in full in Table 3) supported the hypothesis that BPD-PTSD comorbidity would be associated with worse HRQOL than either disorder alone. The diagnostic group variable had a significant main effect (*p* < .01) on each of the eight SF-12v2 scales when all three levels of the diagnostic status variable (BPD-only, PTSD-only, and comorbid) were included. When PTSD-only was contrasted against the comorbid group, the diagnostic group variable had a significant main effect (*p* < .01) on each of the SF-12v2 scales except for Physical Functioning, for which the main effect of diagnostic status was only marginally significant (*p* = .014). When BPD-only was contrasted against the comorbid group, the diagnostic group variable had a significant main effect (*p* < .01) on each of the 8 SF-12v2 scales except for Vitality. Mean SF-12v2 scale scores calculated for each diagnostic group using the SAS SURVEYMEANS procedure (Table 4) indicated that the main effect of diagnostic group can be characterized as an association between BPD-PTSD comorbidity and greater deficits in HRQOL than those seen in PTSD or BPD alone.

**Table 3:** Variance in SF-12v2 scores accounted for by diagnostic status and gender

3-Level Dx Status Models: Comorbid vs. PTSD-only vs. BPD-only				2-Level: Comorbid vs. PTSD-only			2-Level: Comorbid vs. BPD-only		
Scale/Effect	*df*_num_	*F*	*p*	*df*_num_	*F*	*p*	*df*_num_	*F*	*p*
*General Health* (*df*_denom_ = 3690)									
Dx status	2	11.32	**<.0001**	1	22.57	**<.0001**	1	11.56	**.0007**
Gender	1	1.39	.2378	1	1.04	.3090	1	0.31	.5797
*Social Functioning* (*df*_denom_ = 3687)									
Dx status	2	57.43	**<.0001**	1	97.42	**<.0001**	1	12.59	**.0004**
Gender	1	9.28	**.0023**	1	7.80	**.0053**	1	9.76	**.0019**
Dx status*Gender	–	**–**	**–**	1	5.23	.0223	**–**	**–**	**–**
*Role Emotional Functioning* (*df*_denom_ = 3689)									
Dx status	2	56.04	**<.0001**	1	100.79	**<.0001**	1	19.52	**<.0001**
Gender	1	2.59	.1077	1	1.87	.1718	1	2.81	.0942
*Mental Health* (*df*_denom_ = 3688)									
Dx status	2	80.95	**<.0001**	1	119.53	**<.0001**	1	6.94	**.0086**
Gender	1	17.13	**<.0001**	1	16.30	**<.0001**	1	13.35	**.0003**
Dx status*Gender	**–**	**–**	**–**	1	4.38	.0365	**–**	**–**	**–**
*Physical Functioning* (*df*_denom_ = 3691)									
Dx status	2	6.00	**.0025**	1	6.01	.0143	1	12.50	**.0004**
Gender	1	0.21	.6465	1	0.03	.8668	1	0.02	.8874
*Role Physical Functioning* (*df*_denom_ = 3690)									
Dx status	2	9.87	**<.0001**	1	18.87	**<.0001**	1	14.18	**.0002**
Gender	1	0.12	.7280	1	0.01	.9301	1	0.35	.5545
*Vitality* (*df*_denom_ = 3688)									
Dx status	2	19.57	**<.0001**	1	26.74	**<.0001**	1	1.16	.2821
Gender	1	18.50	**<.0001**	1	14.64	**<.0001**	1	15.52	**<.0001**
*Bodily Pain* (*df*_denom_ = 3689)									
Dx status	2	9.53	**<.0001**	1	18.37	**<.0001**	1	13.05	**.0003**
Gender	1	0.05	.8266	1	0.12	.7309	1	0.60	.4393

Results for interaction effects are listed only where *p* < .05; *p*-values less than .01 are bolded

**Table 4:** SF-12v2 Scale means by diagnostic group, by gender, and by diagnostic group*gender

	PTSD-only	BPD-only	Comorbid	Men	Women	PTSD-only		BPD-only		Comorbid	
	(*n* = 3074, *M*_overall_ = 46.64)	(*n* = 483, *M*_overall_ = 44.71)	(*n* = 547, *M*_overall_ = 41.08)	(*n* = 1201, *M*_overall_ = 45.89)	(*n* = 2903, *M*_overall_ = 45.62)	(*n* = 3074, *M*_overall_ = 46.64)		(*n* = 483, *M*_overall_ = 44.71)		(*n* = 547, *M*_overall_ = 41.08)	
	*M* (*SE*)	*M* (*SE*)	*M* (*SE*)	*M* (*SE*)	*M* (*SE*)	*M* (*SE*)		*M* (*SE*)		*M* (*SE*)	
						Men	Women	Men	Women	Men	Women
						(*M* = 46.75)	(*M* = 46.60)	(*M* = 45.29)	(*M* = 44.11)	(*M* = 42.32)	(*M* = 40.43)
General health	46.16 (0.29)	45.00 (0.66)	41.21 (0.73)	44.40 (0.48)	45.90 (0.30)	45.01 (0.54)	46.65 (0.33)	44.55 (0.85)	45.47 (0.83)	40.96 (0.85)	41.33 (0.72)
Social funct.	46.85 (0.27)	42.63 (0.62)	38.31 (0.69)	45.66 (0.42)	45.06 (0.30)	47.14 (0.49)	46.73 (0.31)	43.65 (0.79)	41.57 (0.83)	41.00 (0.71)	36.92 (0.71)
Role emotional funct.	45.05 (0.27)	41.37 (0.57)	36.63 (0.64)	43.64 (0.43)	43.49 (0.29)	45.23 (0.50)	44.98 (0.32)	41.97 (0.78)	40.74 (0.75)	37.92 (0.79)	35.97 (0.58)
Mental health	46.67 (0.25)	41.10 (0.59)	37.71 (0.64)	45.75 (0.41)	44.41 (0.27)	47.73 (0.44)	46.22 (0.28)	42.21 (0.79)	39.95 (0.81)	40.83 (0.76)	36.09 (0.65)
Physical funct.	47.39 (0.27)	49.05 (0.63)	45.51 (0.65)	47.85 (0.43)	47.14 (0.28)	47.89 (0.52)	47.18 (0.31)	49.40 (0.69)	48.69 (0.96)	45.23 (0.74)	45.66 (0.57)
Role physical funct.	46.27 (0.25)	46.38 (0.61)	42.72 (0.60)	45.75 (0.40)	45.89 (0.26)	46.00 (0.49)	46.38 (0.28)	46.66 (0.80)	46.09 (0.86)	43.07 (0.69)	42.54 (0.59)
Vitality	48.56 (0.24)	46.02 (0.51)	44.38 (0.57)	48.80 (0.40)	47.19 (0.25)	49.59 (0.48)	48.13 (0.27)	47.23 (0.68)	44.76 (0.59)	47.11 (0.70)	42.97 (0.51)
Bodily pain	46.17 (0.28)	46.15 (0.66)	42.14 (0.69)	45.27 (0.43)	45.87 (0.30)	45.38 (0.50)	46.50 (0.33)	46.68 (0.78)	45.59 (0.93)	42.09 (0.85)	41.96 (0.72)

#### Main effects of gender

Across the 3-level (including all three levels of the diagnostic status variable) and 2-level (using a two-level diagnosis variable so as to contrast either BPD-only or PTSD-only with the comorbidity) analyses, a main effect of gender (*p* < .01) was seen on three scales: Social Functioning, Mental Health, and Vitality. Mean SF-12v2 scale scores for each gender (Table 4) indicated that these main effects reflect greater deficits for women than for men across all three diagnostic groups in these areas of HRQOL.

#### Diagnostic group-by-gender interactions

In the analyses comparing PTSD-only with the comorbidity, marginally significant diagnostic group-by-gender interactions emerged on the Social Functioning (*p* = .022) and Mental Health (*p* = .037) scales. Mean scale scores (Table 4) indicated that the association of lower functioning with the comorbidity (versus PTSD-only) was greater for women than for men on these two scales.

#### Effects of CSA on HRQOL

Results from the series of SAS SURVEYREG procedures conducted to assess the variance in scores on each SF-12v2 scale accounted for by CSA status, along with gender, diagnostic status, and all 2- and 3-way interactions (Table 5) supported the hypothesis that CSA would be associated with worse HRQOL than no CSA, but that these associations would not be as robust as those between diagnostic status and HRQOL. In analyses including just the CSA status variable and gender as predictors, CSA had a significant main effect (*p* < .01) on each of the eight SF-12v2 scales, at least a marginally significant main effect of gender (*p* < .05) emerged for all scales, and no significant interactions between CSA and gender occurred. Mean scale scores for the CSA vs. no-CSA groups indicated that these main effects of CSA reflected an association between history of CSA and lower HRQOL. However, when CSA status was entered into the models with diagnostic status (three levels: Comorbid, BPD-only, and PTSD-only) and all 2- and 3-way interactions (among diagnosis, CSA, and gender), the main effect of CSA no longer emerged on any of the SF-12v2 scales; the diagnostic group variable maintained a significant main effect (*p* < .01) on all scales; and gender had a significant main effect (*p* < .01) on Mental Health and Vitality.

**Table 5:** Variance in SF-12v2 scores accounted for by CSA and gender (Model 1), and by diagnostic status, CSA, and gender (Model 2)

Model 1				Model 2			
Scale/Effect	*df*_num_	*F*	*p*	Scale/Effect	*df*_num_	*F*	*p*
*General Health* (*df*_denom_ = 33914)				*General Health* (*df*_denom_ = 3668)			
CSA	1	13.94	**.0002**	Dx status	2	10.55	**<.0001**
Gender	1	5.51	.0189	**–**	**–**	**–**	**–**
*Social Funct.* (*df*_denom_ = 33902)				*Social Functioning* (*df*_denom_ = 3665)			
CSA	1	77.82	**<.0001**	Dx status	2	37.58	**<.0001**
Gender	1	5.48	.0193	**–**	**–**	**–**	**–**
*Role Emotional Funct.* (*df*_denom_ = 33902)				*Role Emotional Funct.* (*df*_denom_ = 3667)			
CSA	1	58.18	**<.0001**	Dx status	2	42.63	**<.0001**
Gender	1	14.97	**.0001**	**–**	**–**	**–**	**–**
*Mental Health* (*df*_denom_ = 33898)				*Mental Health* (*df*_denom_ = 3666)			
CSA	1	122.33	**<.0001**	Dx status	2	42.17	**<.0001**
Gender	1	16.43	**<.0001**	Gender	1	8.54	**.0035**
*Physical Funct.* (*df*_denom_ = 33914)				*Physical Funct.* (*df*_denom_ = 3669)			
CSA	1	12.16	**.0005**	Dx status	2	5.69	**.0034**
Gender	1	23.63	**<.0001**	**–**	**–**	**–**	**–**
*Role Physical Funct.* (*df*_denom_ = 33903)				*Role Physical Funct.* (*df*_denom_ = 3668)			
CSA	1	16.04	**<.0001**	Dx status	2	11.15	**<.0001**
Gender	1	23.60	**<.0001**	**–**	**–**	**–**	**–**
*Vitality* (*df*_denom_ = 33901)				*Vitality* (*df*_denom_ = 3666)			
CSA	1	43.85	**<.0001**	Dx status	2	19.65	**<.0001**
Gender	1	38.35	**<.0001**	Gender	1	7.99	**.0047**
*Bodily Pain* (*df*_denom_ = 33902)				*Bodily Pain* (*df*_denom_ = 3667)			
CSA	1	46.22	**<.0001**	Dx status	2	7.90	**.0004**
Gender	1	10.74	**.0011**	**–**	**–**	–	–

Results are listed only for effects where *p* < .05; *p*-values less than .01 are bolded

#### Additive versus synergistic effects of BPD and PTSD

In order to assess whether the effects of BPD-PTSD comorbidity on HRQOL reflected additive effects of the two disorders or the synergistic effect of their interaction, additional SAS SURVEYREG analyses were conducted with BPD and PTSD as separate, dichotomous variables. A marginally significant interaction of BPD and PTSD emerged on the Vitality scale (*p* = .034). Beyond this interaction of interest, these analyses revealed additional interactions: On the Mental Health scale, a marginally significant PTSD-CSA interaction emerged (*p* = .045); and three-way interactions among PTSD, CSA, and gender emerged on Physical Functioning (*p* = .007) and, though only marginally significant, on Role Physical Functioning (*p* = .014). Four-way interactions were pooled, in these analyses, after it was determined that they were not significant. Additionally, when the three-way interactions were pooled for those scales that did not exhibit any significant three-way interactions (i.e., those other than Physical Functioning and Role Physical Functioning), significant BPD-PTSD interactions occurred on the Mental Health and Vitality scales^1^.

### Healthcare usage

Results from the series of SAS SURVEYREG procedures conducted to assess the variance in the healthcare usage variables (number of overnight hospitalizations, days spent in hospital, and number of times treated in a hospital emergency room [ER] in the last 12 months) accounted for by diagnostic status, gender, CSA status, and all 2- and 3-way interactions largely supported the hypothesis that BPD-PTSD comorbidity would be associated with more intensive use of healthcare services than either disorder alone. Diagnostic status had a significant main effect on *times treated in ER*, *F*(2, 3663) = 6.19, *p* < .01, and a marginally significant main effect on *number of days spent in hospital*, *F*(2, 3664) = 3.07, *p* = .046. No other main effects, nor any interactions, achieved significance. The means of these two healthcare usage variables for the comorbid group (*M*_*days*_ = 2.752, *SE* = .563; *M*_*ER*_ *=* 1.188, *SE* = .132) were greater than those for either of the single-disorder groups (BPD-only: *M*_*days*_ = 1.963, *SE* = .355; *M*_*ER*_ *=* .753, *SE* = .089; PTSD-only: *M*_*days*_ = 1.538, *SE* = .158; *M*_*ER*_ *=* .653, *SE* = .035)^2^.

#### Additive versus synergistic effects of BPD and PTSD

When these SAS SURVEYREGs were run with BPD and PTSD as separate variables, no significant interactions of BPD and PTSD emerged, suggesting that the effects of this comorbidity on these healthcare usage variables are better characterized as additive, rather than synergistic^3^.

## Discussion

Results of the present study supported many of the hypotheses. In particular, the finding that those who reported history of CSA had significantly greater odds of being in the comorbid group than either the BPD-only or PTSD-only groups provides empirical, general population-based support for the focus on CSA as an important potential precursor to BPD-PTSD comorbidity. Gender differences in the prediction of BPD-PTSD comorbidity were not as straight-forward as hypothesized—being a woman did not increase the odds of being in the comorbid group versus either of the single disorder groups. However, interesting gender difference findings did emerge: Being a woman significantly increased the odds of being in the PTSD-only group as compared to the BPD-only group and as compared to the comorbid group; and being a woman increased the odds of being in the comorbid group as compared to the BPD-only group. This pattern suggests the possibility that gender difference in PTSD prevalence (which contrasts the relatively gender-balanced BPD prevalence) may contribute to, if not drive, the gender difference in the comorbidity. Looking at the prediction of diagnostic status separately for women and men produced similar patterns of prediction, save for two interesting discrepancies: (1) The association between older age and the comorbidity versus BPD-only was only significant for women. Such a finding reiterates the need for examination of developmental factors to further elucidate this comorbidity. (2) Among those with CSA, the odds of having the comorbidity were significantly greater than the odds of PTSD-only for both genders, but this difference was approximately 3-fold for men and 2-fold for women. This finding suggests that CSA may be an even more specific predictor of BPD-PTSD comorbidity among men than among women.

When age was examined as a predictive factor across the entire sample (i.e., not separated by gender), those in the older age groups had significantly greater odds of being in the PTSD-only group than in the comorbid or BPD-only groups. Such a finding calls attention to the need for further examination of BPD, PTSD, and their comorbidity from a developmental lifespan perspective. Is this age-related finding merely the result of the accumulation of more years, and/or more vulnerable years, in which to potentially experience trauma? Are we seeing the result of some people ‘aging out’ of their BPD diagnoses? Researchers are now looking at the stability of BPD diagnosis and changes in symptomatology later in life, for example, examining the possibility that changes related to personality later in life (e.g., less clinically significant impulsivity) place some individuals who once met criteria for BPD outside of the diagnosis and into a subthreshold or qualitatively distinct category (for a review of relevant findings, see Oltmanns & Balsis [50]). Moreover, changing contexts in later life can alter the impact of personality disorders (PDs) and/or individual symptoms, adding further complexity to the picture of changes in PD pathology across the lifespan [50].

Examination of the prevalence of a broad array of potentially traumatic experiences, from both childhood and adulthood, in our sample offered some additional support for the notion of CSA an important part of the trauma profile associated with BPD-PTSD comorbidity. CSA ranked in the upper range of reported traumas (6^th^ out of 23 types of traumatic experience) in the comorbid group, and some of those five types ranking higher than CSA are arguably relatively inevitable by the time of adulthood (e.g., the unexpected death of a close loved one). Comparing prevalence rates across diagnostic groups and gender was, perhaps, more instructive than rank ordering. The comorbid group had more than double the prevalence rate of CSA than that seen in either of the single-disorder groups. And, lining up with the suggestion made above that CSA may be a more specific predictor of BPD-PTSD comorbidity for men than for women, the percentage of comorbid men (19.14 %) who reported CSA was greater than that of BPD-only men (7.62 %) or PTSD-only men (6.51 %) who reported CSA; and the difference for women across the diagnostic groups was less pronounced (43.42, 28.24, and 24.33 %, respectively). Accordingly, the gender disparity in CSA prevalence rate was much smaller in the comorbid group (19.14 % of men vs. 43.42 % of women) than in either of the single-disorder groups (BPD-only: 7.62 % vs. 28.24 % and PTSD-only: 6.51 % vs. 24.33 %).

In addition to examining traumatic experiences and potential predictive factors associated with BPD-PTSD comorbidity, the present study examined associations between this comorbidity and public health-related dependent variables—specifically, health-related quality of life (HRQOL) and usage of healthcare services. Results of the HRQOL analyses supported the hypothesis that BPD-PTSD comorbidity would be associated with particular deficits. An association was found between BPD-PTSD comorbidity and lower HRQOL than seen in BPD alone or PTSD alone, generally echoing the findings of prior clinical (e.g., [15, 16]) and community (e.g., [2]) studies. Furthermore, our findings extend prior findings to the context of lifetime comorbidity, suggesting that deficits in HRQOL associated with BPD-PTSD comorbidity may not be limited to cases involving current or past year diagnosis of PTSD.

Results of additional analyses representing the comorbidity as the interaction of BPD and PTSD indicated that most of the associations of this comorbidity with lower HRQOL reflect additive rather than synergistic effects of BPD and PTSD. Some BPD-PTSD interactions, however, did emerge. Depending on the specification of these models (i.e., pooling or not pooling of non-significant three-way interactions) and use of lifetime versus current PTSD, either marginally or fully significant interactions of BPD and PTSD emerged on the Vitality and Mental Health scales. Thus, the possibility of synergistic effects of BPD and PTSD on HRQOL should not be ruled out of consideration, in particular with regard to the Vitality and Mental Health subareas of HRQOL.

As hypothesized, childhood sexual abuse (CSA) also showed an association with lower HRQOL, and this association was not as robust as that between the comorbidity and lower HRQOL. CSA may, in fact, have a lasting impact on the lives of individuals with BPD, PTSD, or both; but the deficits in HRQOL associated with BPD-PTSD comorbidity cannot be explained away based on this, albeit impactful, childhood trauma. Gender also exhibited significant main effects on many of the HRQOL scales. Women tended to show significantly lower HRQOL. Additionally, in the comparison of those with PTSD-only versus BPD-PTSD comorbidity, marginally significant interactions suggest that the association between the comorbidity and deficits on the Social Functioning and Mental Health scales may be stronger for women than for men.

Echoing findings of previous clinical studies (e.g., [15, 16]), results of the present study suggest an association between BPD-PTSD comorbidity and patterns of greater healthcare usage, namely greater frequency of treatment in a hospital ER and a trend toward more days spent in the hospital. Our supplementary *current* PTSD comorbidity analyses of healthcare usage also reflected the association of this comorbidity with greater usage of ER care, but not days spent in the hospital.

Given the overlap between analyses in the present study and those reported by Pagura et al. [2]—using the same data, but differing in diagnostic and analytic approach—we conducted an informal comparison of findings. Several differences stand out, including differences in prevalence and co-occurrence as well as differences in the pattern of HRQOL results. As expected, with the use of more stringent diagnostic rules for BPD in the present study, the composition of the diagnostic groups changed fundamentally. Whereas Pagura et al. found that 30.2 % of individuals diagnosed with BPD were also diagnosed with PTSD, and 24.2 % of individuals diagnosed with PTSD were also diagnosed with BPD; the present study found that 53.11 % of those who met criteria for BPD also met criteria for lifetime PTSD, and 14.69 % of those who met for PTSD also met for BPD.

In analyses addressing HRQOL, the present study’s findings diverged from Pagura et al. [2] with regard to the pattern of results for the physical, but not mental, SF-12v2 scales. In Pagura et al., individuals with PTSD-only and the comorbidity had significantly lower physical scale scores than those with BPD-only; but the comorbid diagnosis was not associated with significantly lower scores than PTSD-only on these scales. In the primary analyses of the present study (i.e., using lifetime diagnosis of PTSD) and the majority of the supplementary analyses (i.e., using current PTSD diagnosis, to be more comparable with the analyses of Pagura et al.), the comorbidity was associated with significantly lower SF-12v2 scores than either single disorder group on both the mental and physical scales. Additionally, whereas the results of Pagura et al. indicated no significant interactions between the comorbidity and gender (using a less stringent *p* < .05 cutoff for significance) for HRQOL, the present study found marginally significant (*p* < .05) interactions suggestive of a more detrimental impact of lifetime BPD-PTSD comorbidity on the Social Functioning and Mental Health scales for women than for men, when contrasting the comorbidity against PTSD-only.

Additionally, a supplementary analysis was conducted in the present study to replicate Pagura et al.’s [2] analysis comparing BPD and PTSD symptom item counts across the diagnostic groups. Though the difference we found between the comorbid and BPD-only groups was of a notably smaller magnitude, our results do replicate the rank ordering of symptom item endorsement found by Pagura et al., such that the comorbid group, on average, endorsed more BPD and PTSD symptom items than either single-disorder group. Pagura et al. concluded that their finding indicated an impact of BPD-PTSD comorbidity on central features of each individual disorder—something not found in a number of prior studies (e.g., [15, 16])—and that this discrepant finding arose from the differences between their large, community sample and the small, clinical samples used in prior studies. Though the finding of the present study generally supports this notion of the impact of this comorbidity on core features of the disorders, further examination across different types of samples, and with more thorough measures of symptom expression and severity, will be necessary in order to firmly support such conclusions.

### Limitations

Several methodological limitations in the present study merit consideration. Reliance on retrospective self-report, most notably in the report of trauma experiences, as always, must be regarded with caution. Additionally, some (e.g., [51]) criticize the NESARC for its use of PD interview items that were not drawn directly from a traditional, validated Axis II diagnostic instrument. However, no single PD interview has been shown to be superior to others; and these other interviews disagree with each other at times [44]. Thus, although notable, it is not clear whether this is a significant limitation.

Diverging from the pure epidemiological approach of isolating disorders under investigation via statistical controls (e.g., for sociodemographics and other comorbid diagnoses), the present study conducted analyses without additional statistical controls. There are myriad pros and cons associated with controlling for such factors. The present study adopted the stance that overly controlling for other factors could reduce the meaning of results such as to represent patterns of disorder that are rarely seen in real life (e.g., BPD-PTSD comorbidity with no other Axis I or II disorder). Thus, rather than use statistical controls, the present study examined sociodemographic variables via descriptive analyses and integrated these variables, as appropriate, into central analyses (e.g., inclusion of the age group and gender variables in the logistic regressions and subsequent separation of the logistic regressions by gender).

The absence of analyses examining specific parameters of childhood sexual abuse (CSA) reflects another important limitation of the present study. A substantial body of findings suggests that specific parameters of CSA (e.g., age at onset, number of abusers/incidents, duration of abusive relationship) may influence associations between CSA and psychopathology; and findings relevant to BPD and PTSD, in particular, highlight the potential predictive nature of such parameters [15, 52]. Unfortunately, the NESARC data provide little information relevant to such parameters of CSA, specifying only *age at onset* of sexual abuse/assault, *frequency* of sexual abuse/assault throughout the lifespan, and *age at most recent event* of sexual abuse/assault. Contrary to expectations based on the literature and extant findings, supplementary analyses conducted in the present study to address whether *age at onset* and/or *frequency* of abuse/assault were predictive of comorbid BPD + PTSD status indicated that these parameters were not significantly predictive.

Finally, the slightly high prevalence of lifetime PTSD suggested by our results, and general concerns regarding implementation of diagnostic criteria, prompted further examination of PTSD prevalence and the NESARC diagnostic data for trauma and PTSD. The widely cited National Comorbidity Survey [53] and its replication [54], reported lifetime PTSD prevalence estimates of 7.8 and 6.8 %, respectively; but across varied epidemiologic studies, prevalence estimates range widely (i.e., from roughly 1 to 11 % [55]). Residing at the high end of this range, our 9.48 % estimate may raise some questions regarding the NESARC PTSD diagnosis. As described above, the diagnostic interview employed in the NESARC closely followed the *DSM-IV* criteria for diagnosis of PTSD. The *DSM-IV* version of PTSD Criterion A allows for a relatively broad range of potentially traumatic event types, emphasizing the individual’s subjective experience of these events in determination of whether a potentially traumatic event has occurred (e.g., [56]). Supplementary analysis of the ‘worst stressful events’ endorsed by the PTSD-positive (PTSD+; *n* = 3621) individuals in the present study indicated *unexpected death of someone close* (882 individuals, 24.26 % of the PTSD+ individuals), *sexual abuse/assault* (519, 14.33 %), and *serious life-threatening illness/accident/injury to someone close to you* (470, 12.98 %) as the three most prevalent. None of the other event categories captured more than 6 %, with *beaten up by spouse/romantic partner* ranking fourth most prevalent and capturing 5.52 %. Of all event types coded as a ‘most stressful life experiences’ in the NESARC data, those designed to capture indirect trauma are the most potentially concerning, as some of these event categories—including the first and third most prevalent in our PTSD+ sample—conceivably could capture events that are not congruent with the *DSM-5* revision of Criterion A [10] or conventional objective notions of trauma. Both an *unexpected death of someone close* and a *serious, life-threatening illness/accident/injury of someone close* are events that transpire multiple times throughout most individuals’ lives. Whereas the revised *DSM-5* Criterion A specifies that such indirect experiences of actual or threatened death must be violent or accidental, *DSM-IV* (and thus NESARC) did not make this specification, instead relying on respondents’ subjective reports of fear, helplessness, or horror.

## Conclusions

Overall, findings from the present study help to generalize those from prior BPD-PTSD comorbidity studies to the spectrum of pathology present in the general U.S. population and to the context of lifetime comorbidity. Our findings reflect an important association between CSA and this comorbidity in the general U.S. population, offering support for the focus on this trauma type as an important, but not necessary, etiologic factor. With regard to public health-related outcomes associated with this comorbidity, deficits in HRQOL and increased use of intensive healthcare services, as seen in prior studies, emphasize this comorbidity as a source of greater suffering and public health burden than either BPD or PTSD alone. Further, our findings suggest that the associations of this comorbidity with greater burden of illness are not limited to cases of current or past-year PTSD comorbidity. The present findings argue for the consideration of lifetime comorbidity in clinical, research, and policy-related decisions regarding the study and treatment of these disorders. The differential impact of these disorders occurring alone versus in comorbid form highlights the importance of diagnosing both BPD and PTSD and attending to lifetime comorbidity, despite potential remission of PTSD symptoms. Thus, although specific points of contrast emerged between our findings and those of the prior NESARC-based study by Pagura et al. [2], our overarching conclusions generally extend these authors’ conclusions regarding the non-redundance of BPD and PTSD and the particular burden of this comorbidity—not only to the context of lifetime comorbidity, but also according to the parameters of diagnostic groups defined using a more conventional diagnostic algorithm for BPD. Additionally, the present findings suggest several important future directions, including further investigation of discrepant findings across different types of clinical and community samples, longitudinal investigation of trajectories of HRQOL during and after remission from full PTSD among individuals with BPD, and further investigation of patterns of association across genders and age groups.

## Footnotes

1 Repeating the HRQOL analyses using current rather than lifetime diagnosis of PTSD (i.e., the comorbid and PTSD-only groups contained only individuals who met criteria for current PTSD, and individuals with past but not current PTSD were shifted to the BPD-only group) yielded a very similar pattern of results. Supplementary analyses assessing the additive versus synergistic effects of BPD and current PTSD on HRQOL also yielded results that were very similar to those reported above. These results are available from the corresponding author.

2 Supplementary analyses employing current diagnosis of PTSD highlighted times treated in ER as the only dependent variable on which the diagnostic group variable had a marginally significant (p = .0113) main effect. The diagnostic group means again reflected higher usage of ER treatment in the comorbid group (comorbid: *M*_*ER*_ *=* 1.283, *SE* = .156; BPD-only: *M*_*ER*_ *=* .750, *SE* = .078; PTSD-only: *M*_*ER*_ *=* .720, *SE* = .048).

3 Supplementary analyses employing current diagnosis of PTSD replicated this pattern of results: No significant interactions of BPD and current PTSD emerged.
